# ADME studies and preliminary safety pharmacology of LDT5, a lead compound for the treatment of benign prostatic hyperplasia

**DOI:** 10.1590/1414-431X20165542

**Published:** 2016-11-24

**Authors:** F. Noël, J.B. Nascimento-Viana, L.A.S. Romeiro, R.O. Silva, L.F.N. Lemes, A.S. Oliveira, T.B.S. Giorno, P.D. Fernandes, C.L.M. Silva

**Affiliations:** 1 Laboratório de Farmacologia Bioquímica e Molecular, Instituto de Ciências Biomédicas, Universidade Federal do Rio de Janeiro, Rio de Janeiro, RJ, Brasil; 2 Departamento de Farmácia, Faculdade de Ciências da Saúde, Universidade de Brasília, Brasília, DF, Brasil; 3 Laboratório de Desenvolvimento de Estratégias Terapêuticas, Universidade Católica de Brasília, Brasília, DF, Brasil; 4 Laboratório de Farmacologia da Dor e Inflamação, Instituto de Ciências Biomédicas, Universidade Federal do Rio de Janeiro, Rio de Janeiro, RJ, Brasil

**Keywords:** Benign prostatic hyperplasia, ADME, Safety, Permeability, CYP, Preclinical development

## Abstract

This study aimed to estimate the absorption, distribution, metabolism and excretion (ADME) properties and safety of LDT5, a lead compound for oral treatment of benign prostatic hyperplasia that has previously been characterized as a multi-target antagonist of α_1A_-, α_1D_-adrenoceptors and 5-HT_1A_ receptors. The preclinical characterization of this compound comprised the evaluation of its *in vitro* properties, including plasma, microsomal and hepatocytes stability, cytochrome P450 metabolism and inhibition, plasma protein binding, and permeability using MDCK-MDR1 cells. De-risking and preliminary safety pharmacology assays were performed through screening of 44 off-target receptors and *in vivo* tests in mice (rota-rod and single dose toxicity). LDT5 is stable in rat and human plasma, human liver microsomes and hepatocytes, but unstable in rat liver microsomes and hepatocytes (half-life of 11 min). LDT5 is highly permeable across the MDCK-MDR1 monolayer (P_app_ ∼32×10^-6^ cm/s), indicating good intestinal absorption and putative brain penetration. LDT5 is not extensively protein-bound and is a substrate of human CYP2D6 and CYP2C19 but not of CYP3A4 (half-life >60 min), and did not significantly influence the activities of any of the human cytochrome P450 isoforms screened. LDT5 was considered safe albeit new studies are necessary to rule out putative central adverse effects through D_2_, 5-HT_1A_ and 5-HT_2B_ receptors, after chronic use. This work highlights the drug-likeness properties of LDT5 and supports its further preclinical development.

## Introduction

As the population ages in developed countries and also in countries with fast-growing economies such as the so-called BRICS, which account for around 42% of the world population, the impact of progressive diseases is a global concern. As such, benign prostatic hyperplasia (BPH) has a considerable impact on the health and quality of life of a large portion of aging men (1).

BPH is a nonmalignant progressive enlargement of the prostate and is characterized mainly by stromal hyperplasia in the periurethral transition zone and sympathetic autonomic nervous system hyperactivity (2). BPH pathophysiology involves a static component caused by prostatic enlargement and a dynamic component due to an increased smooth muscle contraction, and both contribute to the lower urinary tract symptoms suggestive of BPH (LUTS/BPH). LUTS/BPH is characterized by hesitancy, weak urinary stream, frequent urination and urgency. Moreover, prostatic enlargement may also be associated with discomfort, and sexual dysfunction (2).

The current drug market includes drugs to treat LUTS/BPH, such as the classical α_1_-adrenoceptor blockers and the more recently approved phosphodiesterase 5 inhibitor (PDE5-I) tadalafil, and drugs to shrink the prostate, such as 5-alpha-reductase inhibitors (5-ARI). The α_1_-adrenoceptor blockers, currently recommended as first-line therapies for moderate to severe LUTS/BPH (3), reduce symptoms by relaxing the prostatic smooth muscle (4). High affinity antagonists for the α_1A_ subtype (tamsulosin, silodosin, alfuzosin) are generally preferred due to less postural hypotension albeit not presenting higher efficacy than the older non-selective α_1_-adrenoceptor blockers terazosin and doxazosin, which were recently pointed as the most effective drugs in a recent meta-analysis (1). It should be noted that the selection of the most appropriate drug for treating LUTS/BPH in older persons (>65 years) could be different (5). On the other hand, the two 5-ARI drugs approved for prostate shrinkage (finasteride and dutasteride) exert antiandrogenic action that may cause adverse sexual effects.

Some years ago we initiated a radical innovation program aiming to obtain a first-in-class drug, as a single compound with efficacy on both urinary symptoms and prostatic hyperplasia (prevention/slowing of disease progression). First, we showed that the *N*1-(2-methoxyphenyl)-*N*4-piperazine moiety confers affinity for α_1A_-, α_1D_-adrenoceptors and 5-HT_1A_ receptors (6). Then, we reported that one of the derivatives, LDT5, was a multi-target compound designed to exert both prostatic relaxation and antiproliferative actions in BPH through action at three different receptor targets: antagonism at α_1A_-adrenoceptor providing mechanism of action (MOA) for treating LUTS while antagonism at the α_1D_-adrenoceptor and 5-HT_1A_ receptor would provide MOA for preventing human prostatic stromal hyperplasia (7). As a result, such compound would have potential clinical use either as monotherapy in first stages of BPH or after treatment, to shrink the prostate (e.g., with a 5-ARI).

In our previous paper (7), we detailed the *in vitro* and *in vivo* pharmacodynamics properties of LDT5 that support our hypothesis of a multi-target antagonism for a dual effect in BPH treatment. Here, we report data addressing the drug-likeness and safety of our elected compound. Indeed, early *in vitro* ADME (absorption, distribution, metabolism and excretion) screening is essential in a drug discovery process for verifying if a drug candidate has desirable drug metabolism and pharmacokinetics (PK) profiles that warrant further preclinical development (8,9). At the same time, preliminary tests for de-risking a lead compound are also required in order to decrease the high attrition rate observed during the drug discovery and development process (10,11). Present results indicate that LDT5 has no safety concern and that its drug-likeness properties support its further preclinical development.

## Material and Methods

### LDT5 synthesis

**Figure 1:**
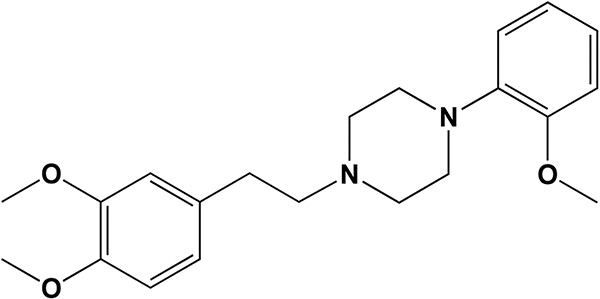
2-D structure of LDT5 in its base form.

LDT5 (1-(2-methoxyphenyl)-4-[2-(3,4-dimethoxyphenyl)ethyl]piperazine monohydrochloride) was synthesized and characterized by spectroscopy as previously described (7). Figure 1 provides the 2-D structure of LDT5 in its base form.

### Animals

All experiments were conducted in compliance with the ethical standards of the Universidade Federal do Rio de Janeiro (licenses DFBCICB011 and DFBCICB015-04/16) and with the recommendations of the Committee on Care and Use of Laboratory Animals of the National Research Council of the United States.

### Physicochemical properties and drug-likeness

The molecular properties of LDT5 were calculated using the ACD/Percepta software, version 14.0.0 (Build 2254), PhysChem module (Advanced Chemistry Development, Inc., Canada).

### *In vitro* ADME studies

These studies were performed at Advinus Therapeutics Limited (India).

### Solubility at pH 7.4

This assay was performed as part of the routine *in vitro* PK assays, even knowing that LDT5 (a monohydrochloride salt) was highly water soluble. The study was performed in a 96-deep well plate by spiking 10 µL of working stock solutions to 990 µL of 50 mM sodium phosphate buffer, pH 7.4. After 2 h, the plate was centrifuged at 1,000 *g* for 20 min at room temperature and aliquots were withdrawn from the supernatant and diluted 1:1 with acetonitrile for analysis by a validated LC-MS/MS detection method using labetalol as an internal standard, a BDS Hypersil Phenyl (150*4.6, 5 µm) column and a mobile phase composed of 5 mM ammonium formate:acetonitrile (40:60, % v/v) with 0.05% formic acid. An API 4000 mass spectrometer (Applied Biosystems/MDS SCIEX, Canada) was used for detection in a positive ionization mode and with the following MRM transitions: 357.4→165.2 and 329.2→162 for LDT5 and labetolol, respectively.

### Plasma protein binding and stability

Spiked human plasma was placed in the donor compartment and phosphate buffer in the acceptor compartment of a HTDialysis 96-well apparatus (USA). The plate was sealed and incubated at 37°C for 6 h at 60 rpm under 5% carbon dioxide atmosphere. Diclofenac (5 µM) was used as a positive control and its mean fraction unbound was 0.28%. The remaining LDT5 spiked plasma was incubated at 37°C for 6 h to assess the stability of LDT5.

### *In vitro* metabolism in rat and human liver microsomes and hepatocytes

The intrinsic clearance in rat and human liver microsomes (0.5 mg/mL protein) and hepatocytes (1 million cells/mL) was conducted at 0.5 µM of LDT5, diclofenac and cocktail of positive control (diclofenac, 7-hydroxycoumarin and testosterone). Microsomes: LDT5 and diclofenac were incubated along with rat (male, Sprague Dawley) and human (mixed, Caucasian) liver microsomes (pooled, from XenoTech, LLC, USA) and co-factor (NADPH, 2 mM). Samples were collected up to 30 min in acetonitrile. NADPH-free control reactions were performed in a similar manner. Hepatocytes: LDT5 and cocktail of positive control were incubated along with rat and human hepatocytes. Samples were collected up to 120 min in acetonitrile. The remaining percent of compound was determined by considering peak area ratio in the 0 min sample as 100%. The first order decay equation was used to estimate half-life using GraphPad Prism® software (USA).

### Permeability on MDCK-MDR1

The *in vitro* apparent permeability was determined across MDCK-MDR1 cell monolayer PreadyPort plate (Readycell, Spain). The cell culture medium contained glucose (1.8 g), HEPES (2.98 g), 10% fetal bovine serum (50 mL), L-glutamine (5 mL), 100 U/mL; 0.1 mg/mL Pen/Strep (5 mL) and 1X MEM non-essential amino acid solution, added to 435 mL of Dulbecco’s modified eagle medium. On the day of the experiment, TEER values were measured for each well on the plate. Buffer containing 1% DMSO was added to the apical compartment (0.25 mL) and to the basolateral compartment (0.75 mL) and placed in an incubator maintained at 37°C and 5% CO_2_ for 30 min. The bioanalysis of LDT5 and quinidine (positive control) was performed using LC-MS/MS.

Cumulative amount of LDT5 (Q) transported at each time point (30, 60, 90, and 129 min) was plotted as a function of time. The slope corresponds to the rate of appearance of test item (dQ/dt) in the receiver compartment and the apparent permeability (P_app_) was calculated using the formula: P_app_ = (dQ/dt) / AxC_0_, where A=surface area of the membrane and C_0_=initial concentration.

### CYP profiling

The *in vitro* metabolic rate of LDT5 was determined in the presence of purified cytochrome P450 (CYP) 1A2, 2C9, 2C19, 2D6 and 3A4 human isozymes (Cypex, UK). The study was conducted at 0.5 µM LDT5 with 10 pM CYP and 2 mM NADPH in phosphate buffer at 37°C in a 96-deep well plate after pre-incubation for 10 min. LDT5 and CYP specific probe substrates were incubated separately along with buffer, purified CYPs and NADPH. The time-dependent loss of the parent compound was determined using LC-MS/MS detection for estimation of the respective half-lives, through nonlinear regression analysis using the first order decay equation.

### CYP inhibition

The inhibition of CYP 1A2, 2C9, 2C19, 2D6 and 3A4 isozymes by LDT5 was evaluated in human liver microsomes (pooled, from XenoTech, LLC) by monitoring the production of selective metabolites following incubation with probe substrates. For each isozyme, a CYP-specific probe substrate was incubated along with microsomes, 1 mM NADPH and LDT5 up to 100 µM. The reaction plate was incubated at 37°C for time periods specific to each isozyme.

### *In vitro* off-target receptor binding

These assays were performed at Eurofins Cerep-Panlabs (France), according to the SafetyScreen 44 panel, using mainly classical competition binding assays and enzymatic inhibition assays with human recombinant proteins (HEK-293 cells). Experimental conditions for each of these 44 assays are available at the Cerep web page: http://www.cerep.fr/cerep/users/pages/catalog/profiles/DetailProfile.asp?profile=2646. Dopaminergic D_2_ receptor binding using rat striatum synaptosomes were performed as previously described (12).

### Preliminary safety pharmacology

*Rota-rod*. Swiss Webster adult male mice (around 40 g) were trained for 1 day and 1 h before the test. Only mice that stayed at least 1 min without falling down were selected. LDT5 (10 µg/kg) or saline were administered *iv* through the orbital plexus (50 μL) of 6 mice per group. After 3, 10, and 30 min, the number of falls during a 4-min observation period was recorded together with the latency for each fall.

*Acute toxicity test*. Six Swiss Webster female mice (25–30 g) per condition received a single dose (100 μg/kg, *ip*) of LDT5. During the first hour and 2, 4, and 8 h after administration as well as daily until the 14th day, different parameters were observed according to the method described earlier (13). The body temperature was registered by an anal probe before, and 30 and 60 min after drug administration.

## Results

### Physicochemical properties and drug likeness

In order to assess the drug likeness of our lead compound LDT5, we first describe its *in silico* ADME profile, a step that is classically performed early, for hit-to-lead progression (14). As shown in Table 1 (15–19), LDT5 met all the criteria of the rule of five as well as the target values for polar surface area and number of rotatable bonds. These two properties reflect the ability to permeate cells and the conformational flexibility of a molecule, respectively (18). This table also includes two ligand efficiency metrics (LE and LipE) for the three target receptors of LDT5. The values of lipophilic efficiency (LipE) within the target values (15) are important since this parameter sets consistent expectations for ligand efficiency regardless of molecular weight or relative potency (16).

**Table:**
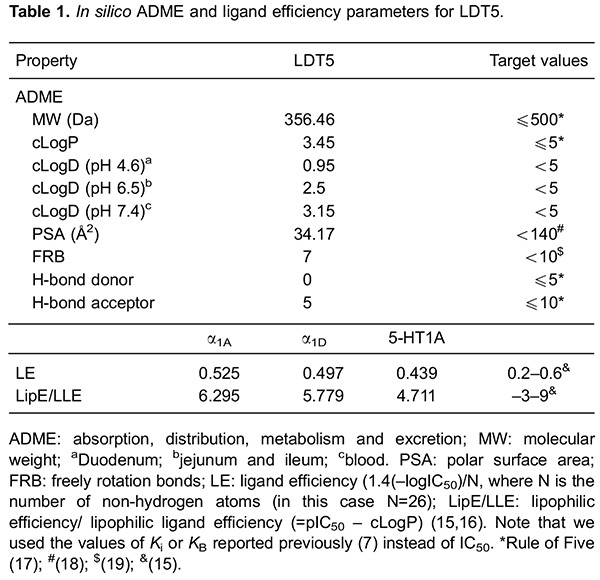

### *In vitro* ADME studies

LDT5 was soluble up to 100 µM, the highest concentration tested, in sodium phosphate buffer, pH 7.4. As assessed by equilibrium dialysis, binding of LDT5 to plasma proteins was intermediate at 10 µM [means±SD values were 80±2 and 73±1% (n=6) in rat and human plasma, respectively].

The *in vitro* apparent permeability of LDT5 was determined across MDCK-MDR1 cell monolayer, using LC-MS/MS analysis of the samples. Our results indicated that LDT5 is highly permeable (P_app_=32.2±1.1×10^-6^ cm/s) for apical to basolateral transport and (P_app_=32.4±1.1×10^-6^ cm/s) for the reverse transport (n=3). Moreover, LDT5 is not a substrate of glycoprotein P (P-gp) (efflux ratio=1) contrary to quinidine (efflux ratio=197.5) P_app_=0.19±0.05×10^-6^ cm/s for apical to basolateral transport and 39.5±2.8×10^-6^ cm/s for the reverse transport (n=3), used as a classical substrate of P-gp for indication of proper functioning of MDCK-MDR1 cells and monolayer integrity.

LDT5 was stable in rat and human plasma at 37°C with recovery of 87±4 and 91±4% (n=6), respectively, after 6 h incubation.

**Figure 2:**
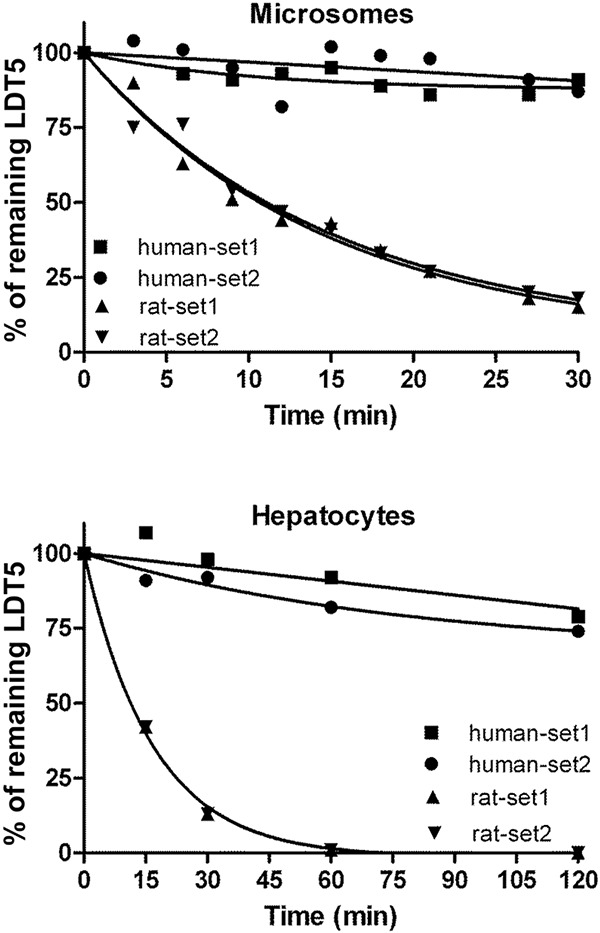
Time-dependent loss of LDT5 in rat and human liver microsomes (*top*) and in rat and human hepatocytes (*bottom*). LDT5 (0.5 µM) was incubated in the presence of liver microsomes (0.5 mg/mL) or hepatocytes (1 million cells/mL). Individual values were obtained from two separate experiments. The fitted curves were obtained by nonlinear regression analysis using the first order decay equation.

The intrinsic clearance of LDT5 in rat and human was estimated *in vitro* using liver microsomes and hepatocytes. The concentration of LDT5 used in these assays (0.5 µM) was chosen according to the typically low concentrations (1–5 µM) of test compound used for incubation to ensure the linearity of enzymatic reaction, but higher than its putative clinically relevant concentration since it is 250–2500 times higher than the affinity for its main target receptors (7). From these data (Figure 2), we can conclude that LDT5 is stable in human liver microsomes (half-life >30 min; intrinsic clearance <0.6 mL·min^-1^·g liver^-1^) and hepatocytes (half-life 120 min; intrinsic clearance <0.2; 0.22 mL·min^-1^·g liver^-1^). On the other hand, LDT5 is unstable in rat liver microsomes (half-life of 10 min; intrinsic clearance = 6.4; 6.7 mL·min^-1^·g liver^-1^) and hepatocytes (half-life of 11 min; intrinsic clearance >2 mL·min^-1^·g liver^-1^).

We then investigated the involvement of CYPs in the metabolism of LDT5, using the human CYP1A2, 2C9, 2C19, 2D6 and 3A4 isozymes. To summarize our results (Table 2), LDT5 was found to be a substrate of CYP2D6 with a half-life of 5 min and CYP2C19 with a half-life of 20 min. In all other tested CYPs, including CYP3A4, the half-life was greater than 60 min.

**Table:**
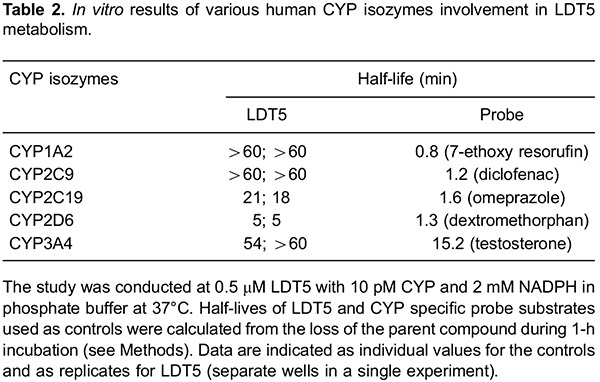

As early assessment of putative drug-drug interaction through CYP inhibition is becoming routine in drug development projects, we estimated the capacity of LDT5 to inhibit the main important CYP450 isoforms (1A2, 2C9, 2C19, 2D6 and 3A4) in human liver microsomes (Table 3). The IC_50_ values for LDT5 against five CYP isozymes were greater than the highest concentration assessed in this assay (100 µM). As a conclusion, LDT5 is not an inhibitor of tested CYPs under these experimental conditions.

**Table:**
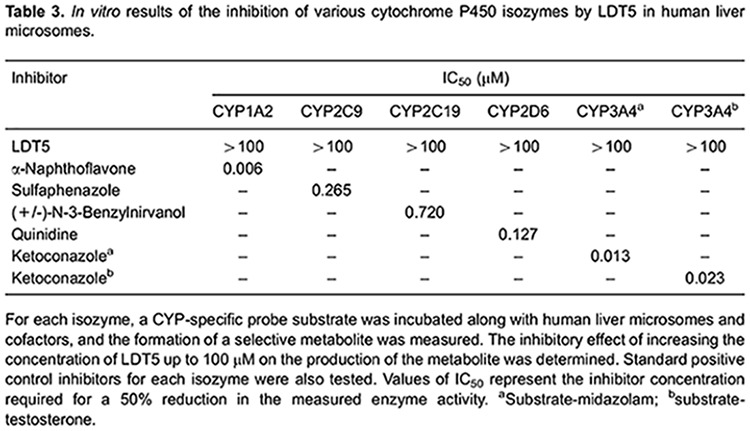

### De-risking: *in vitro* off-target receptor binding

We decided to screen our compound for a panel of 44 targets selected based on the recommendations of researchers from four major pharmaceutical companies (10) since *s*uch a profiling panel (SafetyScreen 44, Eurofins Cerep-Panlabs) can provide an early identification of significant off-target interactions.

One way to quantify “pharmacological promiscuity”, i.e., the property of a compound to have pharmacological activity at multiple targets, is to calculate the percentage of off-targets at which the compound displayed ≥30% activity at a concentration of 10 µM (20 [Bibr B21]). In our case, we did a similar analysis, but using the threshold of 50% activity at 1 µM, in a way similar to the alternative used by Peters et al. (20) when focusing on the more relevant “strong” off-target activity (defined by at least 90% activity at a concentration of 10 µM). The rationale for our proposal is that both formulas are equivalent since we can calculate that a drug with an IC_50_ of 1 µM for example would exhibit both 90% activity at 10 µM and 50% activity at 1 µM (based on the equation for a classical bimolecular reaction, i.e. using the classical “concentration-effect relationship”).

Of 42 BPH off-targets assayed, 7 deserved our attention for their effect at the screening concentration. For dopaminergic D_2_ and 5-HT_2B_ receptors, only rough estimates of *K*_i_ were possible (Table 4). For the dopaminergic D_2_ receptor, we were able to perform a whole competition curve in our validated conditions using rat striatum synaptosomes (12), in order to have a precise value of *K*_i_. In these conditions, the *K*_i_ was 0.10 µM, around 10 times higher than the rough estimate shown in Table 4. Due to the high affinity of LDT5 for its main therapeutic target (*K*_B_=0.18 nM, α_1A_-adrenoceptor), the selectivity of LDT5 estimated by the ratio *K*_i_ off-target/*K*_i_ α_1A_, and thus safety, was considered large enough for all these 7 putative off-targets.

**Table:**
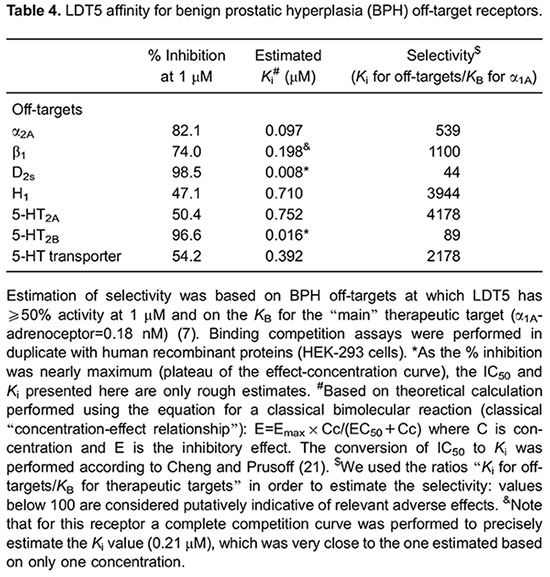

### Preliminary safety pharmacology

*Rota-rod test*. As a preliminary central nervous system (CNS) safety pharmacology, we decided to test LDT5 in the unspecific rota-rod test, an assay that has traditionally been used in mice and rats for motor coordination assessment (22). Such test could also detect drug-induced drowsiness such as caused by alteration of CNS dopaminergic D_2_ receptor function. Considering the time of observation (10 min) both groups showed a similar profile. Two mice treated with LDT5 (10 µg/kg, *iv*) fell once, one after 3 min and the other after 10 min of drug administration. This did not seem different from what occurred with the control mice. These preliminary results indicate that LDT5 has no acute effect on mice motor coordination when administered at a relevant dose by the *iv* route of administration.

*In vivo assessment of acute toxicity*. We also performed a preliminary assessment of acute toxicity by observing different behavioral properties during 14 days after a very high single dose of LDT5 (100 μg/kg *ip*). Indeed, as we had no plasma exposure data (to be obtained later), we used a dose that is 1000 times higher than the *iv* ED_50_ for blocking the phenylephrine-induced increase in intraurethral pressure (7). At the dose studied, LDT5 produced no lethality neither temperature change after 30 min (35.5±0.2°C) and 60 min administration (35.17±0.31°C) as compared to basal (35.5±0.27°C) or saline (35.5±0.22°C) (6 animals per condition). Furthermore, no changes in behavior were observed according to the following parameters: state of attention care and welfare (general appearance, irritability), motor coordination (general activity, response to touch, constriction response of the tail, abdominal contraction, walking, and reflex stiffness), muscle tone, central nervous system activity (tremors, convulsions, hyperactivity, sedation, hypnosis, and anesthesia), the autonomic nervous system activity (lacrimation, defecation, urination, pilo-erection, hypothermia, and breathing), water and food intake.

## Discussion

In an attempt to mitigate the risks inherent to academic preclinical drug discovery projects, we decided to follow some hallmarks of the “product-oriented” organizations, such as the pharmaceutical industries (11). After having described the pharmacodynamics properties of a first series of *N*1-(2-methoxyphenyl)-*N*4-piperazines (6), we then synthesized and characterized a small series of derivatives (7). At that point, the challenge was “to select and advance one or two compounds with properties that are predictive of good efficacy and safety in humans” (23). We elected one of these compounds, LDT5, as our lead compound based on its *in vitro* and *in vivo* effects in relevant pharmacodynamics models aiming to treat LUTS/BPH and reduce BPH progression (7). Here we discussed other of its properties, predictive of good pharmacokinetics and safety profile.

When compared to target values based on successful drugs, LDT5 offered physicochemical properties *in silico* predictive of drugability, such as good oral absorption, due to high solubility (monohydrochloride salt) and high permeability (class 1 of biopharmaceutical classification system) (24), later confirmed by experimental *in vitro* assays. Note that LDT5 was transformed into a monohydrochloride salt not only to increase its water solubility but also (mainly) to avoid formation of N-oxides (which occurs by reaction with oxygen from the air). This is important because the N-oxidation would prevent the protonation of the amine, which is essential for interaction of LDT5 with the carboxylate residue of the receptor. On the other hand, based on a PSA target value (25) for passing the blood brain barrier (BBB) of 90 Å^2^, we can expect that LDT5 would be able to cross the BBB so that de-risking for central effects would be necessary. This expectation was further supported by the permeability assay using MDCK-MDR1 cells indicating that LDT5 was not a substrate of P-gp. The binding of LDT5 to human plasma proteins is not a problem not only because it is considered as intermediate (<85%), but also because even a high proportion of protein binding is no longer considered as a problem since many of the top 100 most prescribed drugs have greater than 98% plasma protein binding (26). As a comparison, tamsulosin has been reported (27) to bind extensively to human plasma proteins (around 99%). The intrinsic clearance of LDT5 in rat and human were estimated using liver microsomes and hepatocytes, which are now considered the gold standards (28). Intrinsic clearance reflects the inherent ability of the liver to metabolize the drug, i.e., in the absence of flow limitation (29). Based on the half-lives measured in our experiments, LDT5 was considered stable in human liver microsomes (and hepatocytes) but unstable in rat liver microsomes (and hepatocytes). Indeed, a compound can be considered to be metabolically unstable when the percent remaining at 60 min is less than 20%, when incubated with 1 mg protein/mL of liver microsomes (30). Such species difference is putatively due to the fact that LDT5 is metabolized by CYP2C19 and CYP2D6 and the substrate specificities are largely different between human and animal isoforms for the CYP2C isozymes (31). Such results (low intrinsic clearance in human models) should be considered as very good for clinical translation, but some concern could arise for pharmacokinetic-toxicokinetic studies if using rats, as usually done in drug development programs.

With respect to the CYP phenotyping, the positive aspects are that LDT5 is not a substrate of human CYP3A4 and is metabolized by more than one enzyme, properties that decrease the risk of drug-drug interaction (DDI) in good accordance with the desirable PK profile for a drug candidate for de-risking DDI (8). On the other hand, a negative aspect is that LDT5 is metabolized by CYP isoforms (CYP2D6 and CYP2C19) characterized by high genetic polymorphism, which is a concern. However, this is not a reason for a no-go decision since there are various examples of successful drugs that are substrates of 2C19 (e.g., citalopram, diazepam, omeprazole) or 2D6 (e.g., metoprolol, paroxetine). This point of view is reinforced by the fact that the reference drug for BPH treatment, tamsulosin, is not only extensively metabolized by CYP2D6 but also by CYP3A4 (32), the major source of drug-drug interaction. Another important point with respect to drug interaction is that LDT5 did not inhibit the main human CYPs, all tested in the present study.

It is now well recognized that assessment of the potential adverse effects of hits is needed as early as possible in the drug discovery process in order to reduce safety-related attrition (10). Compounds aimed to act at an aminergic receptor as a therapeutic target, like LDT5, are particularly prone to pharmacological promiscuity (20), reason why we previously estimated the binding of LDT5 to 5-HT_2A_ and α_2A_-adrenoceptors, in binding assays performed with rat native receptors (7). To estimate the putative adverse effects that could arise from binding at BPH off-target receptors, we now screened LDT5 in a large panel of 44 human receptors routinely used in four major pharmaceutical companies (10) and calculated the ratios “*K*_i_ for off-targets/*K*_B_ for therapeutic target” in order to estimate the selectivity. Such approach raised concern for only two receptors (the dopaminergic D_2_ and the 5-HT_2B_ receptors), albeit LDT5 selectivity (*K*_i_ for off-targets/*K*_i_ for α_1A_) for the D2 receptor was very similar to the one of tamsulosin (33). As we could not find data for tamsulosin affinity for a large panel of human receptors, it was not possible to perform a full comparison of drugs selectivity. Even after having estimated a selective ratio of at least 40-fold for the target α_1A_-adrenoceptor *vs* these off-target receptors, we performed a preliminary CNS safety pharmacology assay using the rota-rod test in mice. Although we did not observe any effect of a single *iv* dose of LDT5, we cannot rule out the risks of effects through central dopaminergic D_2_ receptors such as drowsiness or motor locomotion, since we did not investigate the drug brain penetration and accumulation profiles yet. As there was a concern with putative adverse effects through 5-HT receptors, which play a key role in both central and peripheral mechanisms of thermoregulation (34), we measured mice body temperature and no alteration was observed after administration of a high dose of LDT5 (100 µg/kg, *ip*). We also reported the absence of acute toxicity after a single high dose of LDT5 (*ip*) since no alteration of behavior was observed during the 14-day period of observation.

This work highlights the drug-likeness properties and preliminary safety profile of LDT5, a compound that has been previously selected based on *in vitro* and *in vivo* pharmacodynamics properties aiming at oral treatment of LUTS/BPH. We showed that LDT5 is stable in rat and human plasma, human liver microsomes and hepatocytes, but unstable in rat liver microsomes and hepatocytes. LDT5 is highly soluble in water and permeable across the MDCK-MDR1 monolayer, indicating good intestinal absorption, and is not extensively bound to plasma proteins. LDT5 is a substrate of human CYP2D6 and CYP2C19 but not of CYP3A4, did not significantly influence the activities of any of the human cytochrome P450 isoforms screened and did not present any safety concern, at least in our preliminary assays. Thus, the present results support the further preclinical development of LDT5 and illustrate how a public-private partnership is important to put forward academic drug development projects.
